# Systematic and stochastic influences on the performance of the MinION nanopore sequencer across a range of nucleotide bias

**DOI:** 10.1038/s41598-018-21484-w

**Published:** 2018-02-16

**Authors:** Raga Krishnakumar, Anupama Sinha, Sara W. Bird, Harikrishnan Jayamohan, Harrison S. Edwards, Joseph S. Schoeniger, Kamlesh D. Patel, Steven S. Branda, Michael S. Bartsch

**Affiliations:** 1 Systems Biology, Sandia National Laboratories, Livermore, CA USA; 2 Advanced Systems Engineering & Deployment, Sandia National Laboratories, Livermore, CA USA; 3 Biomass Science and Conversion Technology, Sandia National Laboratories, Livermore, CA USA; 4 Biotechnology and Bioengineering, Sandia National Laboratories, Livermore, CA USA; 5 Present Address: uBiome, San Francisco, CA USA; 6 Present Address: Roche Molecular Systems, Pleasanton, CA USA; 7 Present Address: University of Toronto, Toronto, Canada

## Abstract

Emerging sequencing technologies are allowing us to characterize environmental, clinical and laboratory samples with increasing speed and detail, including real-time analysis and interpretation of data. One example of this is being able to rapidly and accurately detect a wide range of pathogenic organisms, both in the clinic and the field. Genomes can have radically different GC content however, such that accurate sequence analysis can be challenging depending upon the technology used. Here, we have characterized the performance of the Oxford MinION nanopore sequencer for detection and evaluation of organisms with a range of genomic nucleotide bias. We have diagnosed the quality of base-calling across individual reads and discovered that the position within the read affects base-calling and quality scores. Finally, we have evaluated the performance of the current state-of-the-art neural network-based MinION basecaller, characterizing its behavior with respect to systemic errors as well as context- and sequence-specific errors. Overall, we present a detailed characterization the capabilities of the MinION in terms of generating high-accuracy sequence data from genomes with a wide range of nucleotide content. This study provides a framework for designing the appropriate experiments that are the likely to lead to accurate and rapid field-forward diagnostics.

## Introduction

Rapid and accurate pathogen detection is of critical importance in healthcare, both in the clinic as well as in low-resource field settings. Protocols based on current second-generation sequencing technologies, although highly accurate, often require extensive sample preparation work, as well as considerable time for both sequencing and data analysis, before a meaningful result can be obtained. In addition, these sequencing technologies can exhibit bias when applied to GC-rich regions of DNA, primarily due to reduced sequence complexity but also as a result of polymerase chain reaction (PCR) bias during amplification steps^1,2^. Therefore, the application of sequencing to broad-spectrum pathogen diagnostics requires technologies and methods that combine speed with accuracy that is independent of genome composition, particularly with respect to GC content.

The last few years have seen the emergence of third-generation sequencing technologies aiming to achieve an unprecedented combination of speed, accuracy, and flexibility. These include single-molecule real-time sequencing (SMRT) and nanopore techniques that enable highly-parallelized, amplification-free sequencing of individual nucleic acid molecules^3–7^. These approaches allow for incoming data to be processed in real-time, a significant benefit to applications in rapid detection and diagnostics^8–10^. Another feature of these technologies is their ability to sequence long reads up to 100 s of kilobases (kb), which is useful both for resolving highly repetitive genome features that confound the assembly of short-read sequencing data and for deciphering nucleotide rearrangements such as RNA splice junctions^11,12^. Already, there are numerous examples of using these third-generation sequencers to identify pathogens in complex clinical samples, with an eye toward real-time diagnostics^9,13–16^. These sequencers also enable detection of nucleic acid modifications (e.g., methylation), such as those involved in post-transcriptional regulation of gene expression^17–19^.

A notable example of third-generation sequencers, the Oxford Nanopore Technologies (ONT) MinION, is a palm-sized nanopore-based sequencer that enables real-time analysis of sequence data on a laptop computer. The translocations of individual DNA strands through the nanopores within the device yield sequence-dependent changes in the measured ionic current through those pores. The raw current data is parsed and reduced to an event-based “squiggle” wherein discrete events reflect the presence of a particular k-mers in the pore. Event-based k-mer calls are made in a moving window, such that each change in the event squiggle ideally indicates a single-base translocation of the strand through the pore. A Student’s T-test is used to determine whether two consecutive events are significantly different. Machine-learning algorithms recently released by ONT (e.g., Metrichor, nanonet, Albacore, etc.) rely on trained neural networks to convert these moving window events into 5-mers, and the consolidation of these overlapping 5-mers is what results in a basecall. The confidence with which these events can be associated with their respective 5-mers is reflected in a per-base log-scale Q-score^20^.

The quality of MinION basecalling is improved by performing what is known as a 2D read, where both the template and complement strands of the DNA molecule are joined by a hairpin loop during library prep and read sequentially, allowing the basecalls for each strand to be correlated to generate a consensus sequence. In contrast, DNA sequenced without a hairpin, whether by design (e.g., when using a 1D rather than 2D library prep) or circumstance (e.g., when ligation of the hairpin in a 2D library prep fails), yields a single-strand template-only sequence known as a 1D read. Recently, ONT has replaced 2D library prep and sequencing with what is called 1D^2^ (“1D-squared”), where instead of a hairpin physically joining the template and complement strand, the complement is tethered in the vicinity of the pore after the template is read, thereby increasing the likelihood that both strands will be consecutively sequenced and enabling template-complement consensus basecalls. While the biochemistry is different, the basecalling algorithms remain very similar to what is used with 1D and 2D reads.

While there have been a number of studies using the MinION to sequence a variety of sample types, including genomic DNA, amplicons, cDNA, and even RNA, systematic studies of sequence-dependent performance and biases (e.g., with respect to GC content) are rare^20,21^. The goal of the present study was to assess the performance of the MinION sequencer and associated algorithms as applied to bacterial genomes of varying degrees of GC content, in order to evaluate its potential utility as a tool for rapid and organism-independent pathogen identification. We show that while overall the MinION and the neural network-based algorithms perform well regardless of GC content, there are a number of stochastic and contextual sources of variation that can reduce basecalling accuracy. Specifically, we show that problems with basecalling accuracy are due to a combination of local stochastic disturbances at the pore during sequencing, and systemic biases for specific nucleotides that are overcome to a certain extent by calling overlapping k-mers. We also find that the basecallers exhibit context-specific variability depending upon degree of GC content. Taken together, these results provide a framework for understanding and better interpreting the results obtained from MinION sequencing, and also point to critical areas of experimental design that could be manipulated in order to address potential biases and take better advantage of the MinION’s capabilities.

## Results and Discussion

### The MinION sequencer generates similar quality data across a range of GC content

We sought to assess the performance of the ONT MinION, and the associated software called MinKNOW, in sequencing bacterial genomes representing a wide range of GC content. We focused on three species at different points on the GC content spectrum: *Escherichia coli* (~51% GC), *Clostridium difficile* (~28% GC), and *Burkholderia thailandensis* (~68% GC). Genomic DNA was isolated from these bacteria using a phenol-chloroform-based extraction method, and the purity of the samples was verified. We used the MinION 2D library preparation kit in combination with the R9 chemistry-based flowcells to sequence the samples. For the *E*. *coli* DNA sample, we also performed a 1D rapid library preparation (which uses tagmentation rather than ligation to add the adapters) for comparison, which proved useful at multiple points in our work. Of note, however, a 2D library will also produce a number of high-quality 1D reads, enabling us to compare the metrics of 1D vs 2D reads within a single 2D experiment.

With each sequencing run, we were able to obtain 50,000–130,000 reads. The differences in read counts are believed to be primarily attributable to flowcell-to-flowcell variability, with lesser contributions from differences in DNA extraction and library preparation; read counts did not vary with GC content in any predictable way. For the purposes of our analysis, we normalized the sequencing data in terms of reads/active pores/time whenever necessary.

**Figure 1:**
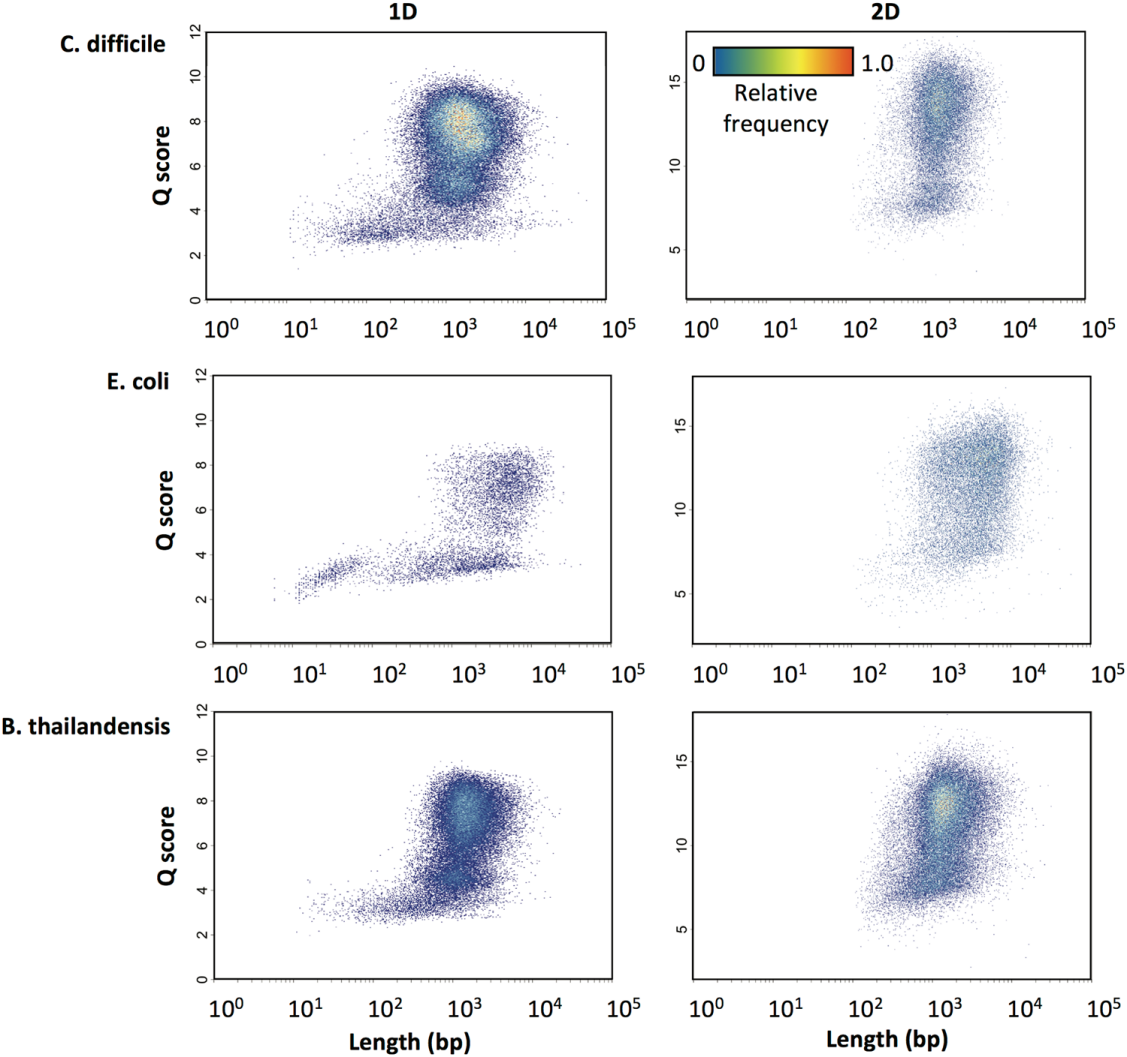
Frequency-normalized 2D histograms of average read Q-score versus read length for 1D and 2D reads (both from 2D sequencing) of *C*. *difficile* (111k reads), *E*. *coli* (54k reads), and *B*. *thailandensis* (131k reads).

For all runs and samples, read lengths generally ranged from 100 bp to 100,000 bp, with an average read length of 1,000–2,000 bp (Fig. 1); this is consistent with previously published reports^22–25^. It is worth noting that recent technological and methodological advances are allowing for sequencing of increasingly longer reads, which will be of paramount importance in applications such as genome assembly.

We noticed that very short reads (≤200 bp) tended to be 1D, and there are a number of possible explanations for this observation. A nick on the first strand, for example, would halt sequencing at the 1D stage. Alternatively, very short DNA templates may be more susceptible to steric interactions during the translocation of the hairpin adapter that prevent proper basecalling of the second strand. It is also possible that, for purposes of generating 2D basecalls, the MinKNOW sequencing software simply ignores reads below a certain size or complement sequence below a certain Q-score. Perhaps an artifact of the operation of the basecaller’s neural net, very short reads also tended to be of low quality (Q-scores of 2–4 for 1D reads) compared to reads ≥ 500 bp in length (Q-scores of 4–10 for 1D reads), but no clear correlation between length and quality was observed above this size range (Fig. 1). Another possibility is that shorter DNA fragments might be more prone to error-inducing stochastic movements during pore translocation due to the Brownian motion of the fluid, whereas longer fragments are less affected, allowing for more consistent movement through the pores. Overall, however, these results confirm that the MinION can generate high-quality sequencing data for all but the shortest DNA fragments, regardless of their GC content.

### MinION-generated high-quality reads map accurately to their reference genomes, with relatively high identity, regardless of GC content

**Figure 2:**
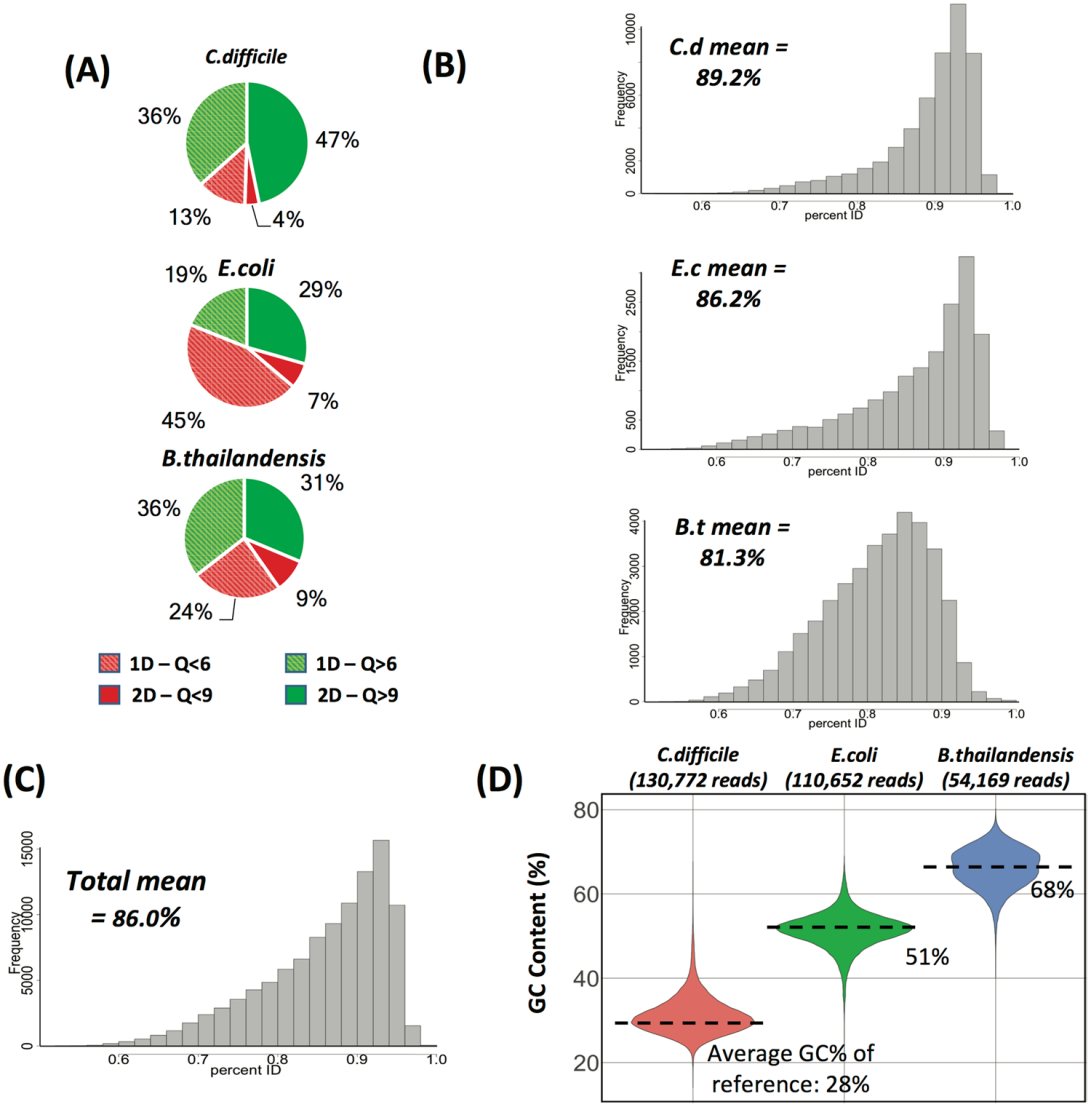
MinION sequencing results for bacterial genomes of varying GC content. (**A**) Pass/fail quality score distributions for 2D-sequencing produced reads (Q > 6 for 1D pass, Q > 9 for 2D pass). Solid slices are 2D reads (template and complement consensus), and striped slices are 1D reads (template only). (**B**) Histogram of percentage identity of reads to reference genome sequences for *C*. *difficile* (top), *E*. *coli* (middle) and *B*. *thailandensis* (bottom). (**C**) Aggregate histogram of percentage identity of all reads to the three reference genome sequences. (**D**) Violin plots showing the percent GC content of reads from MinION sequencing for *C*. *difficile*, *E*. *coli*, and *B*. *thailandensis*. Dotted lines indicate the GC content of the GenBank reference genome sequences.

Given the well documented GC bias associated with amplification-based sequencing library preps^1,2,26,27^, we sought to test whether the amplification-free prep of the MinION was subject to any comparable systemic bias. We observed that the proportion of high-quality versus low-quality reads did not strictly scale with genomic GC content (Fig. 2A). However, we noticed that for high-quality 2D reads (Q-score > 9), only 81% of the *B*. *thailandensis* reads mapped to the reference genome, whereas 99% of the *E*. *coli* reads and 96% of the *C*. *difficile* reads mapped to their respective reference genomes. Moreover, the sequences of the mapped reads deviated from those of the reference genome to a greater degree for *B*. *thailandensis* (81.3% identity) than for *E*. *coli* (89.2% identity) and *C*. *difficile* (86.2% identity) (Fig. 2B–C). Unclear from these results (and beyond the scope of this study) is the degree to which GC biases and errors inherent to the methods used to generate the *B*. *thailandensis* reference genome could account for some or most of the observed disparity in its sequence mapping. Despite these phenomena, however, the overall GC content of the MinION reads did accurately reflect that of their cognate reference genome (Fig. 2D), indicating that there was no apparent issue with reading through GC-rich DNA. In addition, while both for *E*. *coli* and *C*. *difficile* performed better than *B*. *thailandensis*, it is also worth noting that the accuracy for *E*. *coli* was higher than for *C*. *difficile*, suggesting that the relationship between GC-content and accuracy is not necessarily linear. Rather, this might indicate that, while the MinION is able to generate high-quality sequence from DNA templates of varying GC content, there may be certain handicaps that exist at divergent GC compositions due to repetitive or low-complexity regions that are perhaps atypical of the DNA used to train the basecaller’s neural net. Alternately, in this particular case, seemingly lower basecall accuracy could also be attributed in part to a mismatch between the reference used and the particular cell lines sequenced in these experiments.

### Stochastic and systematic biases in base calls by the neural network basecalling algorithm are compensated for by reading the second strand

**Figure 3:**
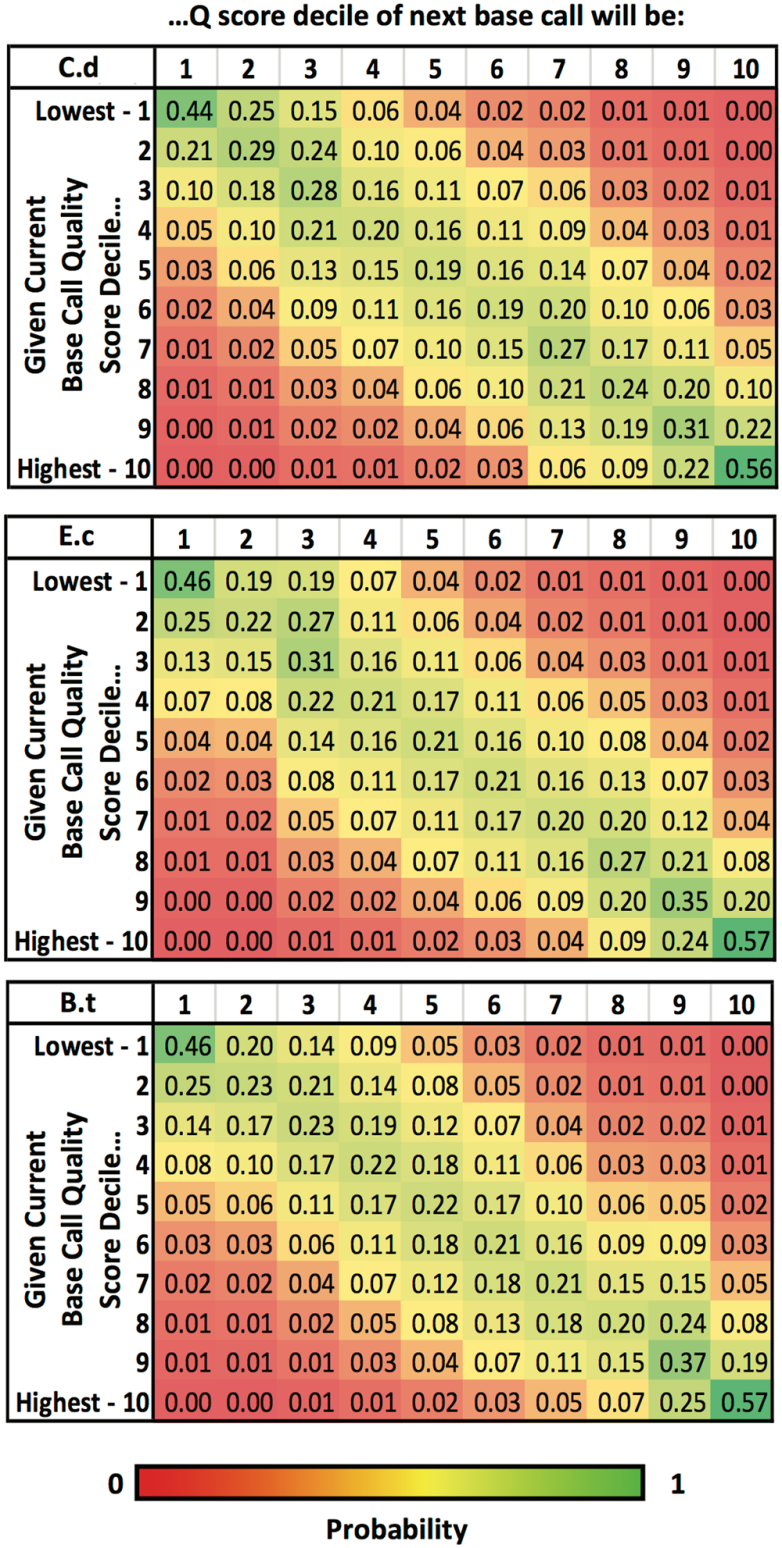
Relatedness of Q-scores across individual basecalls. Average Q-score of each read was broken into individual Q-scores per base, and then divided into ten deciles. Given a Q-score decile for the current base, the probability of the next base having a Q-score in each of the ten deciles was calculated. The probabilities are shown in heat map format for *C*. *difficile* (top), *E*. *coli* (middle), and *B*. *thailandensis (bottom)*.

Recognizing that MinION basecalls are derived by calling k-mers, rather than individual bases, we hypothesized that there could be a significant contextual interdependency of basecall Q-scores. To address this possibility, we obtained individual nucleotide Q-scores for each read (i.e., from the fourth line of each entry in the FASTQ files generated by the basecaller), keeping in mind that the Q-score of an individual base is determined by the consensus basecall confidence score of the overlapping 5-mers used to establish the identity of that base. With this information, we asked: if a given base (referred to as the ‘first base’) was assigned a given Q-score, how (if at all) did its score affect or correlate to the Q-score of the subsequent base (referred to as the ‘second base’)? Because of the wide range of possible Q-scores, we divided the scores into deciles to obtain more useful information about any correlation of scores without overemphasizing stochastic variability. In other words, the Q-scores were discretized into ten bins with an equal number of scores in each bin. We observed that, regardless of the decile in which the Q-score of the first base falls, the best indicator of the Q-score of the second base is the score of the preceding base (Fig. 3). This suggests that the local conditions and stochastic events (e.g., presence of nearby debris, steric interference, electrical noise, etc.) at the time that a base and its neighbors are read are likely the primary sources of variability in basecall quality.

**Figure 4:**
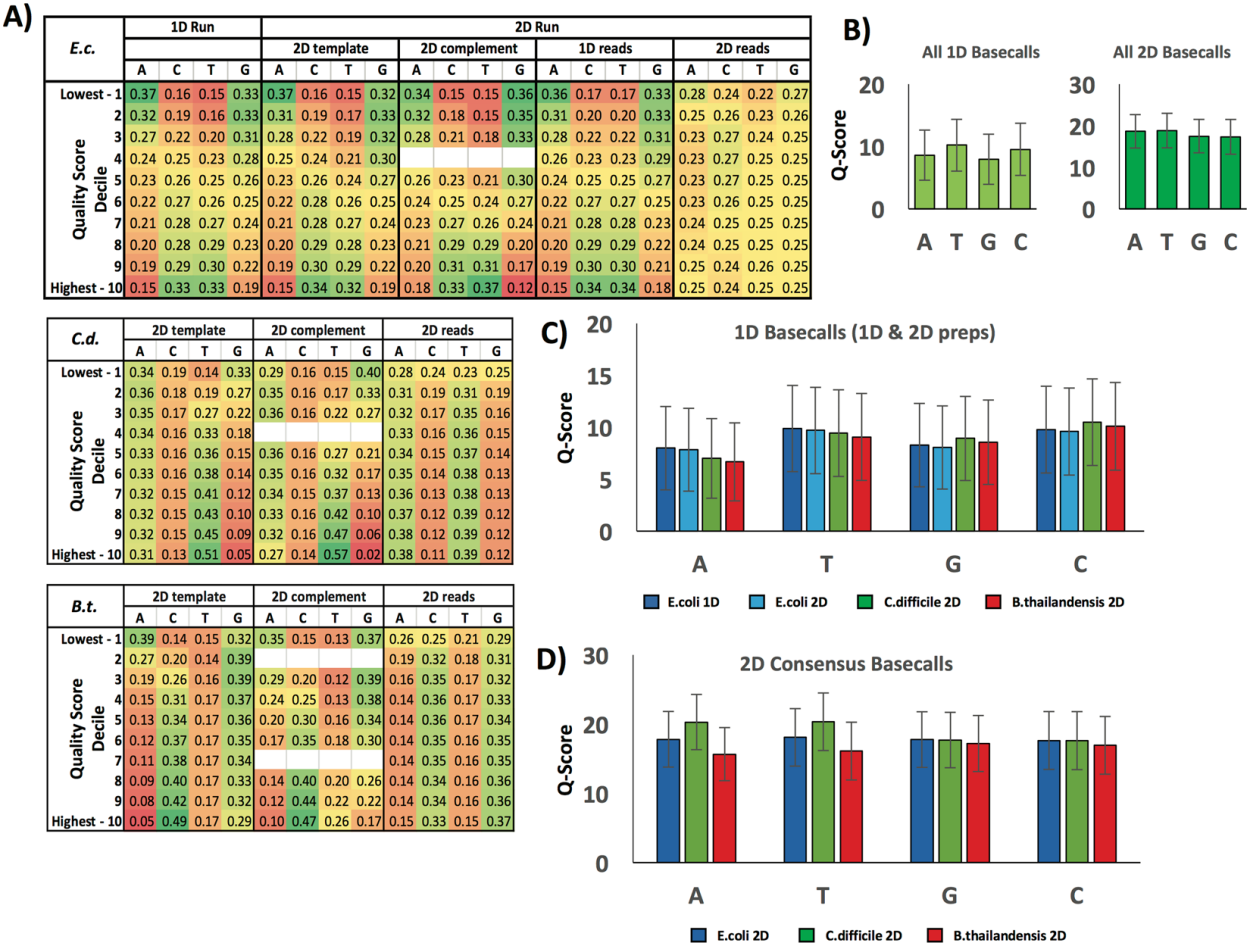
(**A**) Heat map chart showing probability of basecalls in a particular Q-score decile being A, C, T or G. After dividing base Q-scores into deciles (as in Fig. 3), the probability that the base is A, C, T or G, given a Q-score decile was calculated. 1D and 2D data are shown for *E*.*coli (top)*, while only 2D run data are shown for *C*. *difficile (middle)* and *B*. *thailandensis (bottom)*. Note – rounding error may cause some groupings to appear not to sum to 1.00 even though the unrounded numbers do. (**B**) Averages of Q-scores per base for *C*. *difficile*, *E*. *coli* and *B*. *thailandensis* data. (**C**) Average data in (**A**) for 1D and 2D (template only) reads, broken down by organism. (**D**) Data in (**A**) for 2D consensus reads, broken down by organism.

We also wanted to look beyond stochastic influences and investigate whether the underlying sequence had any impact on basecalling, or whether the variation in quality was primarily non-systemic. Examining the *E*. *coli* data (where we expected a relatively even distribution among the four bases), we saw that low-scoring bases were significantly more likely to be A or G and less likely to be T or C, a pattern that was inverted for high-scoring bases (i.e., they were more likely to be called T or C). This was true regardless of whether the run was 1D or 2D, and whether the template or complement strand was being read in the 2D run. In the case of a 2D run, quality deficits in A and G were substantially compensated by the quality advantages of their complements (T and C), yielding a much more uniform probability distribution across the ACTG space for the consensus (template + complement) 2D reads. As a result, 2D basecall probabilities were representative of the underlying *E*. *coli* sequence composition (Fig. 4A – top). We also saw this same basecall bias superimposed on the underlying genome content bias of the *B*. *thailandensis* and *C*. *difficile* sequences (Fig. 4A – middle and bottom). Consequently, the relative abundance of AT base pairs in *C*. *difficile* partly offset the basecaller-induced scarcity of high quality A calls, while further enhancing the prevalence of high quality T; and similarly, in *B*. *thailandensis*, the lower number of high-quality C calls is partially offset by the abundance of GC base pairs. Again, the portion of the probability mismatch attributable to C- and T-favoring basecaller bias was substantially resolved when 2D consensus basecalls were generated (Fig. 4A – middle and bottom), leaving only the underlying AT and GC content biases of the genomes. This observation was confirmed by looking at average Q-scores by base for 1D or 2D reads, either by genome or in aggregate (Fig. 4B–D). The results indicated that, despite the majority of the error being non-systemic, the basecalling algorithm does appear to have certain systemic errors that depend upon the underlying sequence but are substantially resolved for 2D consensus reads. This provides a more detailed explanation for why 2D basecalling accuracy is consistently higher than its 1D counterpart.

### The position of a base within a read has an effect on its quality score (Q-score)

**Figure 5:**
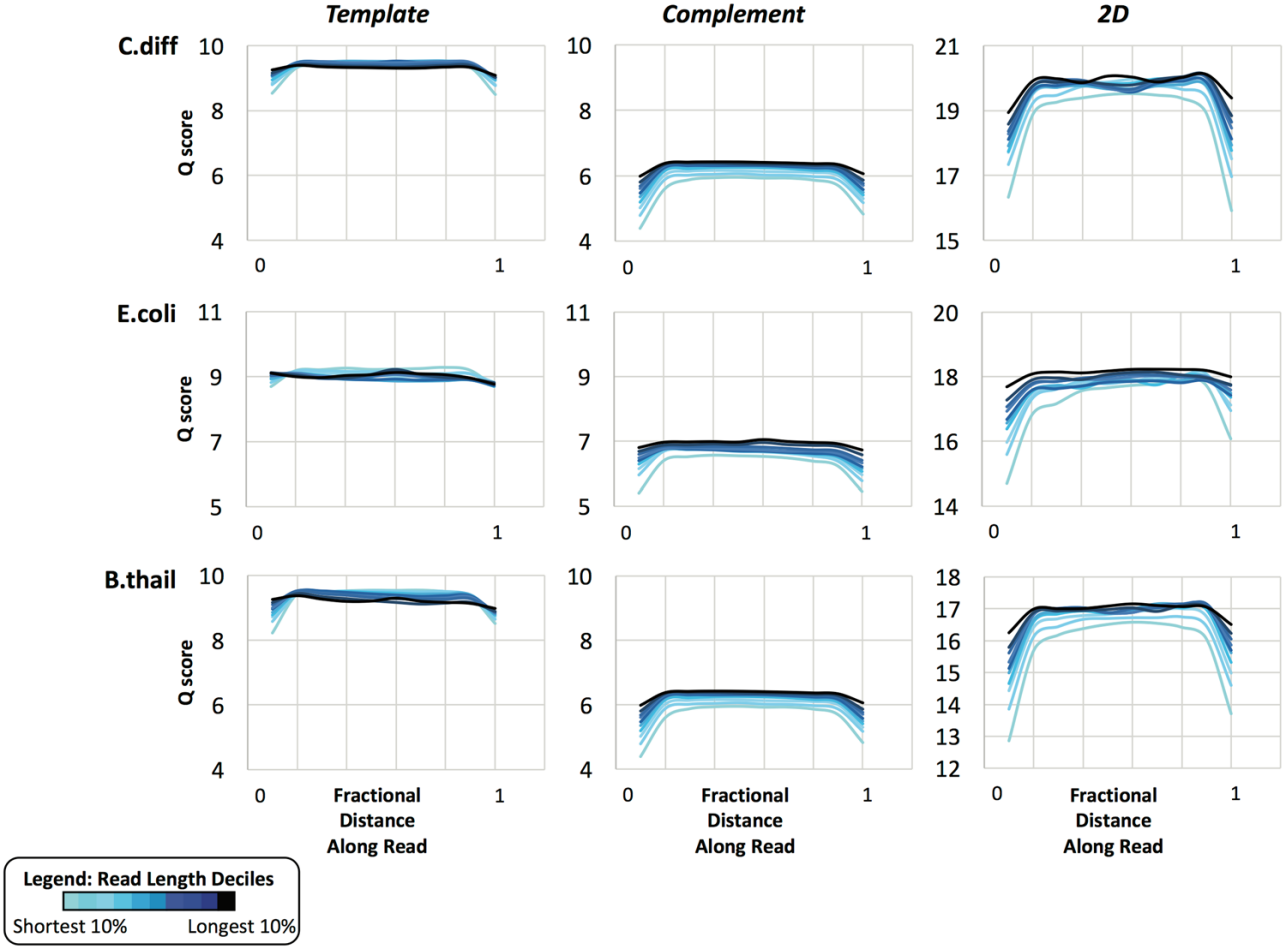
Variation in basecall quality as a function of position within MinION reads. 2D sequenced reads are grouped into deciles based on length. Q-scores as a function of length-normalized position within individual reads are averaged within each length decile group and plotted for template, complement, and 2D consensus basecall results.

While we have seen that the length of a DNA molecule does not, for the most part, affect its average Q-score, we wanted to determine whether individual base Q-scores vary systemically as a function of their position within the read. We divided reads from each MinION run into deciles based on length and then asked what the Q-score was as a function of relative (length-normalized) position within the reads in each decile group (Fig. 5). We found that, on average, the complement strand generated lower Q-scores than did the template strand. Per informal discussions with ONT technical support, this is believed to be due to re-hybridization of the template and complement strands on the downstream side of the pore, which causes the complement strand to spool through the pore at a less consistent rate than is optimal for the basecaller. In addition, for both template and complement strands, the beginning and end of the molecule (at the Y-adapter and hairpin ends, respectively) had notably lower Q-scores compared with the rest of the molecule, an effect that is exacerbated for shorter reads (Fig. 5). This is highly suggestive of a steric or conformational limitation that prevents consistent movement though the pore at either end of the molecule. Again, this observation appears to be independent of GC content, as the effect is observed in each of the three bacterial genomes analyzed (Fig. 5). Also, as observed previously (Fig. 2), the high GC organism (*B*. *thailandensis*) displays lower 2D Q-scores overall compared with *E*. *coli* and *C*. *difficile*. Interestingly, the accuracy with respect to the reference genome appeared to decrease with lower GC content (Fig. 2B), and this correlates with the overall Q-score across the read (i.e. *C*. *difficile* has the highest scores, followed by *E*. *coli*, then *B*. *thailandensis*, Fig. 5).

### K-mer biases in basecalling are affected by GC content of the sequenced genome

**Figure 6:**
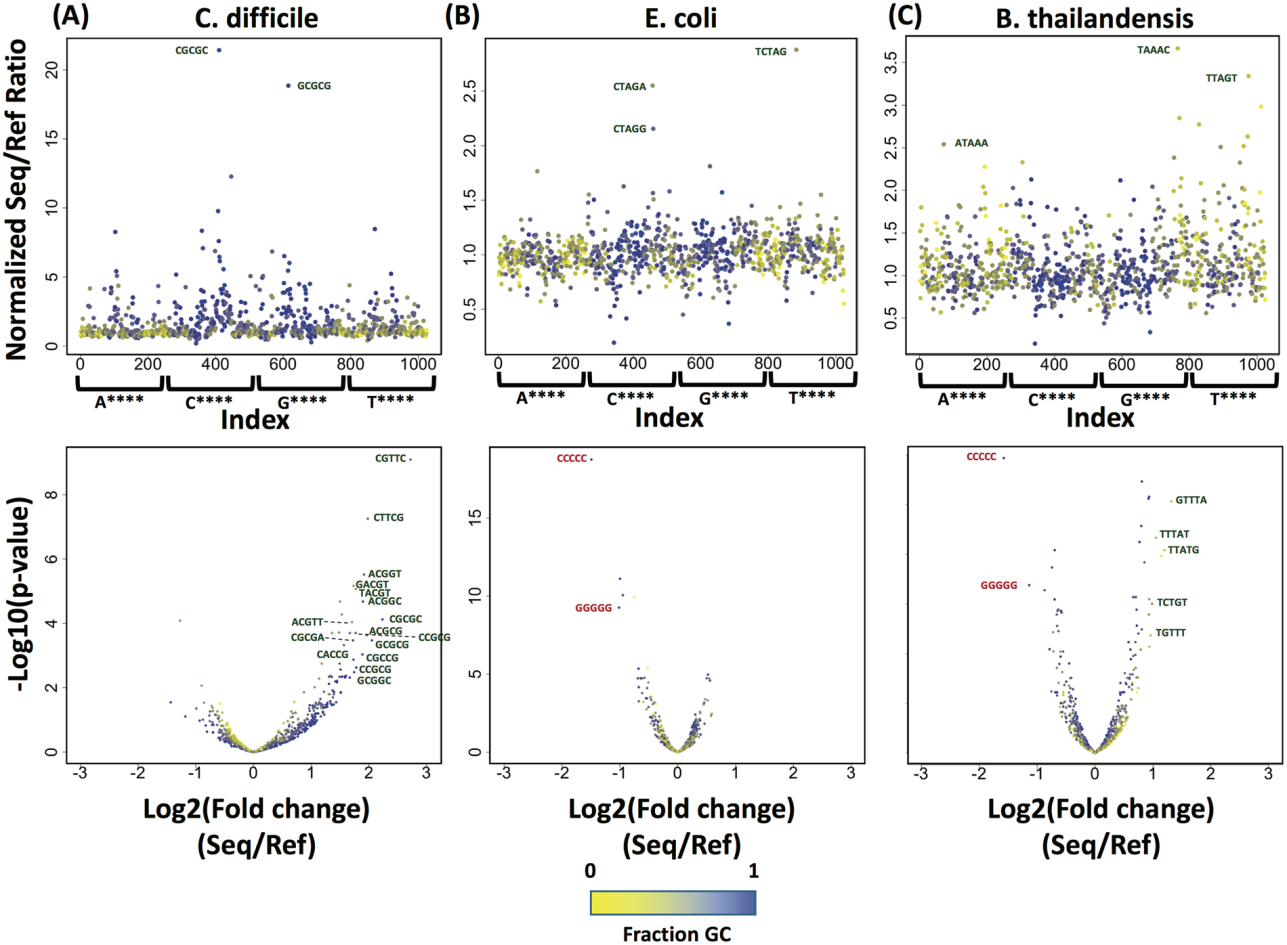
Sequence bias informs k-mer bias during sequencing. Top: Coverage-normalized ratio of occurrence of all possible 5-mers in the MinION sequencing data over the reference genome, for *E*. *coli* (**A**), *B*. *thailandensis* (**B**), and *C*. *difficile* (**C**), arranged alphabetically from AAAAA to TTTTT. Points are colored based on fraction GC content. Bottom: Negative binomial linear regression plotted as p-value versus fold change (i.e., volcano plot). Annotations indicate 5-mers with at least a 2-fold (*E*. *coli*, *B*. *thailandensis*) or 2.5-fold (*C*. *difficile*) difference in representation in the sequencing data versus the reference genome sequence.

In order to characterize the bias of the neural network basecaller in more detail, we compared the incidence of individual k-mers in the sequencing data versus reference genomes for *E*. *coli*, *B*. *thailandensis* and *C*. *difficile*. The neural network algorithm currently uses 5-mers in its model, so we tallied the occurrence of all possible 5-mers in each MinION sequencing data set and its cognate reference genome. We then took the ratio of these counts, and looked for outliers based on Z-score (i.e., number of standard deviations away from the mean, Fig. 6). In all three cases, overrepresentation of certain k-mers in the sequencing data was much more likely than underrepresentation, suggesting that the basecaller makes systematic errors in calling certain k-mers. However, due to the overlap between adjacent k-mer calls, these errors can be corrected almost immediately through consensus calls. We used negative binomial linear regression to identify over-represented or under-represented k-mers in each genome sequence, based on 2 (*C*. *difficile* and *B*. *thailandensis*) or 3 (*E*. *coli*) replicate MinION sequencing runs. Using an adjusted (Benjimini-Hochberg corrected for multiple hypothesis testing) *p*-value threshold of 0.05 for a fit to the regression, we identified 78 to 127 k-mers, of the 1024 k-mers possible, that were significantly over- or under-represented in the MinION sequencing data for each genome (see Table 1 for list). For *B*. *thailandensis* and *E*. *coli*, the vast majority of k-mers were detected at roughly the same levels (within 2-fold variation) in the MinION sequencing data as in the cognate reference genome. These relatively minor discrepancies are likely real, based on their reproducibility, but also are likely resolved by the moving window k-mer basecalling algorithm. For *C*. *difficile*, on the other hand, significantly more variability between the k-mers in the MinION sequencing data versus the reference genome sequence was observed. This was not a result of noisy data, as the two replicate MinION sequencing datasets for *C*. *difficile* were well correlated (Spearman’s coefficient = 0.96, p < 2e-16). One possibility is that, in general, AT-rich 5-mers simply exhibit more variability in the basecall. However, we do not see this variability in the AT-rich 5-mers within the *E*. *coli* and *B*. *thailandensis* genome sequences, so this explanation seems unlikely. Closer examination of the over-represented 5-mers (>2-fold difference) revealed that in the genomes with a strong GC bias (*C*. *difficile* and *B*. *thailandensis*), the miscalled k-mers tended to have the opposite GC content (i.e., GC-rich in *C*. *difficile*, and AT-rich in *B*. *thailandensis*), which is consistent with the logic that a mistake is more likely to contain bases not in the original genome. In *E*. *coli*, on the other hand, the miscalled k-mers were all of similar sequence (CTAG with a flanking base on one or the other side), suggesting a minor systemic error in the basecaller in the event of an evenly-balanced genome.

**Table 1:** Outliers from negative binomial linear regression, cutoff in each case was where Benjamini-Hochberg FDR < *p*-value.

*E.coli*	*B.thail*	*C.diff*
CACGG	AAGGA	CCAAT
CAAGC	ACGAG	CCCCC
TGTGT	ACGAT	GAGAA
GGCCG	ACGGG	GAGAG
TCTTG	CCCCC	GAGTA
CAAGT	CCCGA	GGATT
GTGTG	CCGAG	TCCAA
CGTGT	CCGGA	TTTTA
TCAAG	CGAGA	
TCTGT	CGAGC	
TGTAC	CGATC	
TCTAG	CGATG	
CGCGC	CGATT	
CTAGA	CGCGA	
GTTGT	CGGAG	
CTTCG	CTTGA	
GTTCT	GACGA	
CGCGG	GAGAA	
CTCGG	GAGAT	
GCAAG	GAGCC	
TACGT	GAGCG	
GGCGG	GAGCT	
GCTGT	GAGGA	
CTTGG	GCCCC	
GACAC	GCGAG	
GGTGC	GCTTG	
TCGGC	GGATT	
CAAGG	GGGGG	
TCGTG	TCGAG	
ACAAG		
GCGGC		
GTTGC		
GCCGT		
CGTGC		
CTGTG		
CCTTC		
GCAGC		
GCTGC		
GCACG		
CGGTG		
GCCGG		
CAAGA		
CCTTG		
CCAAG		
GCCTC		
TGTGC		
CGTAC		
TGTGA		
GTGTC		
CGAGC		
CTTCA		
ACCTC		
GTGTA		
GGTAC		
GGTGT		

### A new basecaller, Albacore, results in similar basecalls albeit with higher quality

**Figure 7:**
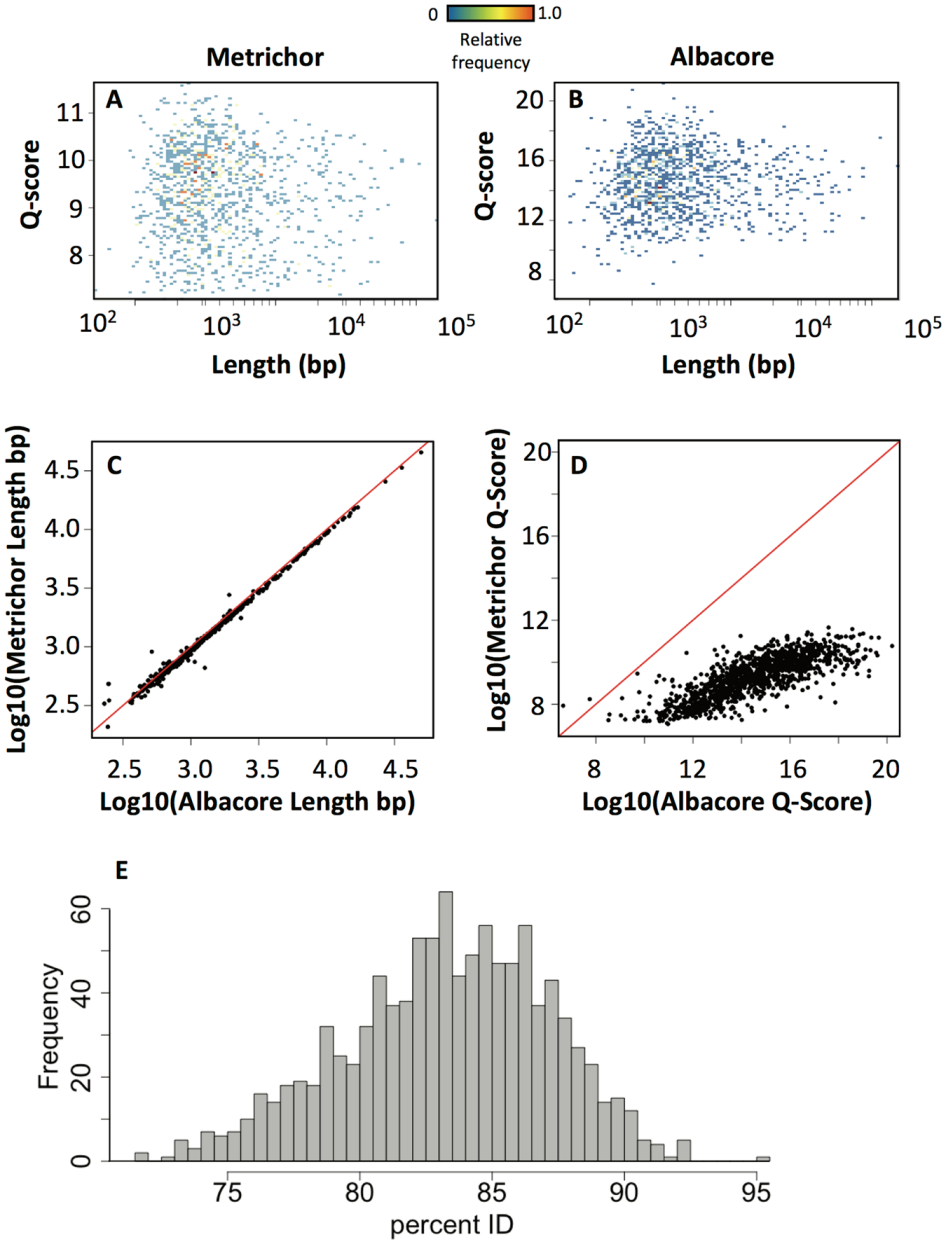
Comparing Metrichor to the new Albacore basecaller using a *E*. *coli* genomic DNA sequenced with the 1D rapid kit. (**A**,**B**) 2D histograms of Q-score versus length for reads basecalled by Metrichor (**A**) and Albacore (**B**). (**C**) Comparison of individual read lengths obtained by Metrichor (y-axis) and Albacore (x-axis). (**D**) Comparison of individual read Q-scores obtained by Metrichor (y-axis) and Albacore (x-axis). (**E**) Histogram of percent identity mapping between individual reads basecalled by Metrichor and by Albacore.

**Figure 8:**
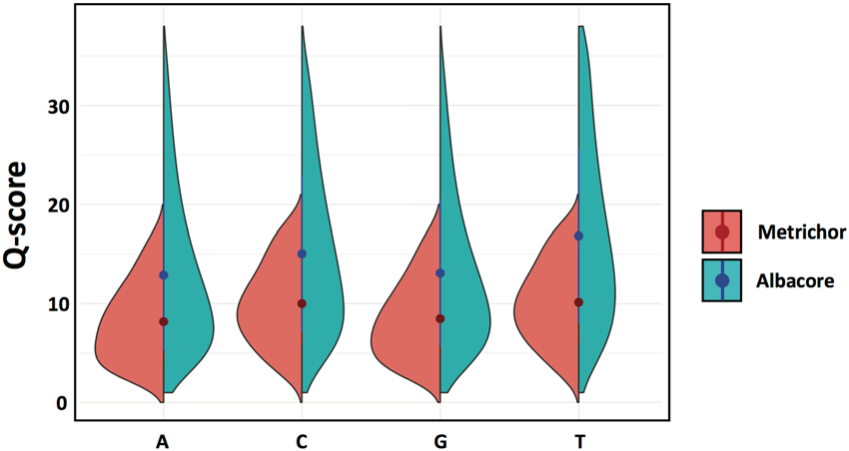
Per base comparison of Metrichor and Albacore. Split violin plots showing the distribution of Q-scores per base for Metrichor (red) and Albacore (blue). The dots represent the means of each split violin, and lines from the center represent the standard deviations.

In the time since we initiated this study, ONT released a new local basecaller called Albacore, which uses a similar algorithm to the latest version of Metrichor (i.e. a neural network). We wanted to compare these two iterations of the basecaller to determine whether there were any significant changes to the algorithm which would affect our conclusions. We basecalled an *E*. *coli* 1D rapid library using both Metrichor and Albacore, and compared the resulting datasets. We found that, overall, Albacore and Metrichor performed similarly in terms of mapping statistics (eg., *E*. *coli* 1D – Metrichor: 94%, Albacore: 97%). The overall lack of obvious correlation between Q-score and length that was observed in Metrichor results was also present in the reads called with Albacore, as evidenced by the similarity of their respective 2D histograms (Fig. 7A,B). Upon closer examination, however, we found that while the lengths of the reads called by Metrichor and Albacore were almost identical, there was a slight tendency for Albacore to call more bases than Metrichor in any given read (Fig. 7C). Additionally, the Albacore reads distributed across a wider range of generally higher Q-scores as compared to the Metrichor reads (Fig. 7D). Nevertheless, the percent identity between reads called using Albacore and Metrichor was centered around 85%, which is similar to the percent identity mapping of reads to the reference genome for both basecallers (Fig. 7E). The improvement in Q-score was also evident on a per-base level for all four bases, with the improvement in the quality of T basecalls was elevated compared with the other three bases (Fig. 8). We do however still see that A and G tend to have lower Q-scores, suggesting a similar base-specific systematic bias as Metrichor (Fig. 8). Taken together, our comparison suggests that Albacore does not produce significantly different basecalls than Metrichor, but does improve the quality of those basecalls.

## Summary and Future Perspectives

In this study, we have evaluated the performance of the ONT MinION device in sequencing bacterial genomes of varying GC content. We observed a wide range of read lengths, but basecall quality did not vary with sequence length except in the case of the shortest reads (<200 nt). However, we found that the beginning and end of each sequenced strand tended to produce basecall data of lower quality, an effect that, while more pronounced in the 2D complement strand, was observed for the template strand as well. Overall, basecall quality appeared to be determined primarily by a combination of non-systemic, context-based errors and systemic base- and k-mer-specific biases, for both basecallers tested (Metrichor and Albacore). These k-mer biases are likely an artifact of the varying degree to which particular k-mers affect nanopore current measurements during strand translocation, but could also be a result of training the neural net algorithms on sequences that underrepresent those particular k-mers. While the former explanation may be fundamental to the operation of the nanopore sensor, the latter could readily be addressed in future releases of the basecalling software by providing the neural net with a more diverse training dataset. In future iterations of the basecaller, it might be possible to train a model that adjusts for this type of systemic error. Our results suggest that mismatches or indels at the beginning or end of a read should be tolerated better than those in the middle of the read, a feature that could be incorporated into nanopore-optimized alignment algorithms.

One possibility for addressing the bias of the neural network basecaller is to develop a correction system, or modify existing read-correction software, based on the nucleotide composition of the reads. For example, if the read in question is GC-rich, any AT-rich k-mer would require a higher bar for validity confirmation (i.e., more support from flanking k-mers). On the one hand, this reduces the independence of individual basecalls, but since the bias with GC-rich or AT-rich sequences is very nucleotide-dependent, implementing a score-based weighting of k-mers could be beneficial on balance.

While improving the scope and precision of basecalling is an essential goal, it raises the issue of accuracy versus speed. Particularly with neural networks, the more complex the network and the larger the number of hidden layers in that network, the longer (exponentially) it takes to train and run it. Indeed, a large number of neural network-based machine learning algorithms require multiprocessing power, often involving graphics processing units (GPUs) or field-programmable gate arrays (FPGAs) instead of central processing units (CPUs). Many machine learning experts are currently working to optimize neural networks without requiring the use of a GPU or similar processing power^28–30^. While training the network is a one-time event, every sequencing run must be basecalled using this network, and with real-time data analysis being one of the major advantages of the MinION, it will be critical in future iterations of the basecaller to improve accuracy while minimizing the number of layers used.

As the speed of nanopore sequencing continues to increase, basecalling algorithms must continue to improve in order to effectively capture and deconvolute ever more rapid and subtle changes in the pore current. Moreover, this must be achieved without significantly increasing the time taken to make basecalls and without compromising basecall accuracy. To date, the majority of use cases for the MinION have been rapid field-based identification of DNA or RNA sequences of interest, which do not necessarily require 100% basecalling accuracy. However, for certain applications, such as genome assembly or single nucleotide polymorphism (SNP) calling, much higher accuracy would be preferable [ours ranged from 85% to 95% (Fig. 2B)]. It is encouraging, however, that the current state of the art in nanopore sequencing has already resulted in a number of complete or near-complete genomes, including the human genome^22,31–35^. While spearheaded by ONT, efforts to this end are also substantially supplemented by the MinION community at large, and resulting performance improvements will further broaden the applicability and enhance the impact of ONT’s nanopore sequencers in both field- and laboratory-based detection and diagnostics.

## Methods

### DNA extraction

Bacterial strains *Escherichia coli* MG1655 and *Burkholderia thailandensis* E264 (ATCC, USA) were grown overnight in LB medium at 37 °C with shaking at 250 rpm, and the bacteria recovered from 1 ml of culture *via* centrifugation (16,000 × *g* for 3 min). *Clostridium difficile* clinical isolate HD-1110-A (a kind gift from Chris Polage, University of California at Davis, CA) was grown on Egg Yolk Agar (Anaerobe Systems, Morgan Hill, CA) using a GasPak anaerobic system (Becton Dickinson, San Jose, CA) at 37 °C for 48 h. All bacteria were lysed using Qiagen lysis buffer (Qiagen, Hilden, Germany) supplemented with proteinase K and RNase A (Thermo Fisher Scientific, Waltham, MA) and incubation for 15–30 min at 50 °C. Genomic DNA (gDNA) was extracted using a phenol-chloroform method, as follows. One volume of phenol:chloroform:isoamyl alcohol (25:24:1) (MilliporeSigma, St. Louis, MO) was added to the lysate, the tube inverted 10–20 times to thoroughly mix, and the mixture centrifuged at 16,000 × *g* for 10 min. The aqueous phase was transferred to a fresh tube, and DNA was precipitated from solution through addition of 0.1 volume of 3 M sodium acetate (pH 5.0) followed by 2.5 volume of 100% ethanol. After mixing well *via* tube inversion and cooling at −20 °C overnight or −80 °C for 1–2 h, the samples were centrifuged at at 16,000 × *g* at 4 °C for 15–30 min. After discarding the supernatants, the gDNA pellets were washed twice with 500 μL of 70% ethanol, left to dry at room temperature for 5–10 min, and then resuspended in nuclease-free water.

### MinION Sequencing

Library preparation for all 2D sequencing runs was performed using the sequencing library preparation kit (SQK-NSK007, Oxford Nanopore Technologies, UK) according to the manufacturer’s instructions with R9 flow cells (MIN106) on a MinION Mk1B sequencer. The end-prep reaction using the NEBNext Ultra II End Repair/dA-tailing module was done at 20 °C for 15 min followed by 10 min at 65 °C. Fragmentation and repair steps were omitted in this library prep. The sequencing library preparation kits were SQK-RAD001 (1D rapid kit) and SQK-NSK007 (2D kit). DNA concentrations were measured using Qubit Fluorimeter (Thermo Fisher Scientific, USA). The flow cells were primed and the library mix was loaded onto the MinION flow cell per manufacturer’s instructions. The 6-hour sequencing protocol was initiated on the MinKNOW software (versions 1.4–1.7).

### Basecalling

We used Metrichor 2.42.2 to basecall our 1D and 2D data. Poretools 0.6.0 was used to convert FAST5 files from Metrichor into FASTQ files for mapping. Statistics were also collected using Poreminion 0.4.4 and fast5tools 0.1^36^. We compared Metrichor with the newest ONT-released basecaller, Albacore 1.2.4, which is available on GitHub.

### Mapping and Percent ID of reads

Reads were mapped using the basecaller BWA under the MEM algorithm^37^ (version XXX). The following settings were used: -k17 -W40 -r10 -A1 -B1 -O1 -E1 -L0 (which are defaulted under the –x pacbio option). We used ‘pacbio’ settings rather than ‘ont2d’ settings, since the latter ended up being too lenient, likely because they were designed for the ONT R7 chemistry, not the current R9 chemistry. We used samtools 0.1.19 and bedtools 2.26.0 for file conversion^38,39^. Percent IDs of each read to the corresponding reference were deduced by parsing the CIGAR string of the SAM files.

### Group-based analysis of Q-scores

Q scores were parsed from FASTQ files by using the SeqIO module of Biopython 1.7. A Hidden Markov Model-based analysis was implemented to determine the transition and emission probabilities of the occurrence of the four nucleotide bases (A, C, T and G) based on Q scores. The scores were divided into deciles to eliminate uninformative complexity in the data.

### K-mer analysis

Using the h5py 2.7.1 module in Python, event data from FAST5 files was parsed for all reads, and the k-mer associated with each detected event was collected and tallied. In parallel, we extracted all possible sequential k-mers from the corresponding genomic regions to which the reads mapped, and tallied those. First, we looked at the ratio of the occurance of the k-mer in the sequencing reads compared with the reference, and determined outliers using a Z-score (number of standard deviations away from the mean). We then compared the distributions of k-mers in the reads to the reference using negative binomial regression, and identified outliers for which the Benjimini-Hochberg false discovery rate was greater than the p-value.

### Data

The fast5 files associated with this work have been deposited on the SRA (Accession PRJNA420504).
